# The Yin and Yang of the Opioid Growth Regulatory System: Focus on Diabetes—The Lorenz E. Zimmerman Tribute Lecture

**DOI:** 10.1155/2016/9703729

**Published:** 2016-09-14

**Authors:** Joseph W. Sassani, Patricia J. Mc Laughlin, Ian S. Zagon

**Affiliations:** ^1^Departments of Ophthalmology and Pathology, Penn State Milton S. Hershey Medical Center, Hershey, PA 17033, USA; ^2^Department of Neural and Behavioral Sciences, The Penn State University College of Medicine, Hershey, PA 17033, USA

## Abstract

The Opioid Growth Regulatory System consists of opioid growth factor (OGF), [Met^5^]-enkephalin, and its unique receptor (OGFr). OGF inhibits cell division when bound to OGFr. Conversely, blockade of the interaction of OGF and OGFr, using the potent, long-acting opioid receptor antagonist, naltrexone (NTX), results in increased DNA synthesis and cell division. The authors have demonstrated both* in vitro* and* in vivo* that the addition of exogenous OGF or an increase in available OGFr decreases corneal epithelial cell division and wound healing. Conversely, blockade of the OGF-OGFr interaction by NTX or a decrease in the production of the OGFr increases corneal epithelial cell division and facilitates corneal epithelial wound healing. The authors also have demonstrated that depressed corneal and cutaneous wound healing, dry eye, and abnormal corneal sensitivity in type 1 and type 2 diabetes in animals can be reversed by OGF-OGFr blockade by NTX. Thus, the function of the Opioid Growth Regulatory System appears to be disordered in diabetic animals, and its function can be restored with NTX treatment. These studies suggest a fundamental role for the Opioid Growth Regulatory System in the pathobiology of diabetic complications and a need for studies to elucidate this role further.

## 1. Introduction

This review focuses on the Opioid Growth Regulatory System and its implications for the pathobiology of diabetes. It was presented, in part, as the Lorenz. E Zimmerman Tribute Lecture at the symposium in Dr. Zimmerman's honor jointly sponsored by the American Academy of Ophthalmology and the American Association of Ophthalmic Oncologists and Pathologists, Chicago, Illinois, October 19, 2014. Dr. Zimmerman was the founder of modern ophthalmic pathology having served at the Armed Forces Institute of Pathology for 52 years. He was mentor to many practicing ophthalmic pathologists and the recipient of numerous national and international honors. He died April 6, 2013, at the age of 92.

## 2. The Opioid Growth Regulatory System

### 2.1. System Overview

**Figure 1:**
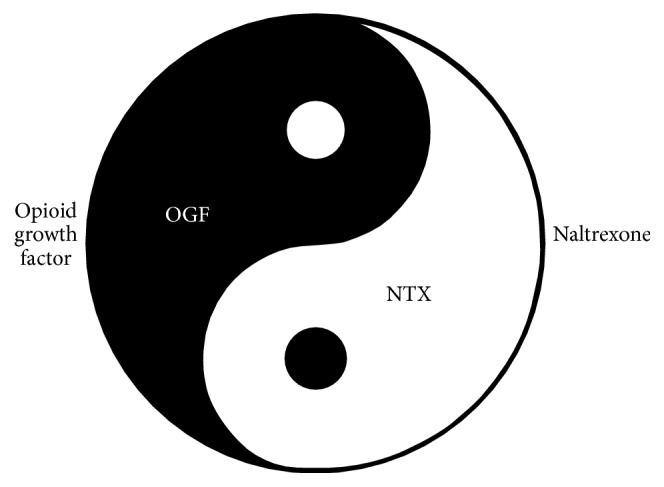
The figure illustrates the two opposing “Yin and Yang” entities: opioid growth factor (OGF), [Met^5^]-enkephalin, which initiates downregulation of cell division, and naltrexone (NTX), which blocks the receptor (OGFr) for OGF resulting in upregulation of cell division and facilitation of epithelial wound healing.

This review highlights the Opioid Growth Regulatory System. In particular, it emphasizes its implications for the pathobiology of diabetic complications including impaired wound healing, abnormal corneal sensitivity, and dry eye.  Figure 1 highlights two of the main and opposing characters in this story: the naturally occurring opioid growth factor (OGF), [Met^5^]-enkephalin, and its pharmacologic antagonist, naltrexone (NTX). This figure is the basis for the title of this review.

### 2.2. Roles of Endogenous Opioids

There are many endogenous opioids. They bind to specific receptors and they perform various biologic functions including analgesia, cardiovascular control, respiration, behavior, learning and memory, emotion, and cell division and growth. This review focuses on the latter function of regulation of growth and cell division by the Opioid Growth Regulatory System. This system, also called the OGF-OGFr axis, is comprised of two major components: opioid growth factor (OGF) itself and its specific “opioid growth factor receptor” (OGFr).

### 2.3. Opioid Growth Factor and Its Receptor

Opioid growth factor, chemically [Met^5^]-enkephalin, is a naturally occurring opioid. It is a pentapeptide with the sequence Tyr-Gly-Gly-Phe-Met. Its action is to depress cell division when bound to the other key component of the Opioid Growth Regulatory System, the specific opioid receptor for OGF, not surprisingly termed OGFr. OGF is potent and reversible and is species and tissue nonspecific.

The OGFr has been cloned and sequenced in the human, rat, and mouse. It has no resemblance to classic opioid receptors. Its specific gene locus is known. OGF bound to this specific receptor is the only such opioid that has an effect on cell division.

OGF is tonically produced so that usually its level in tissues is neither maximized nor minimized. As a result of this characteristic, manipulation of the Opioid Growth Regulatory System, either by the addition of exogenous OGF or by blocking its receptor, can decrease or increase cell division, respectively.

OGF usually is produced in an autocrine or paracrine manner, meaning that it is manufactured by the cells that will be modulated by it or by their neighbors. Nevertheless, systemic levels may be of importance for diabetic complications [1, 2]. It specifically targets cell proliferation.

The Opioid Growth Regulatory System is truly an ancient cellular regulatory mechanism that has been conserved from bacteria to humans [3, 4]. This system can modulate growth and development in embryologic, normally dividing, healing, and even neoplastic tissues (basically, any cell that has the potential to divide). The authors' observations suggest that it does not “overdrive” cell division in tissues that have attained contact inhibition of cell division. It does not alter apoptosis, necrosis, or differentiation.

### 2.4. Opioid Growth Regulatory System Mechanism of Action

When OGF is bound to its specific receptor, OGFr, cell division is suppressed.

**Figure 2:**
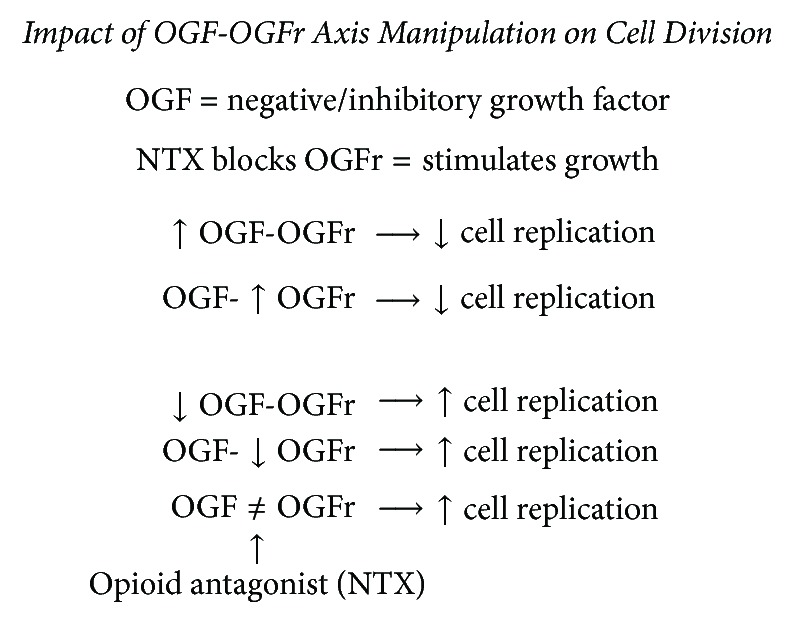
Ways in which the relationship between OGF, OGFr, and NTX can be manipulated to impact cell division.

Figure 2 demonstrates several ways in which the relationship between OGF and its receptor can be manipulated to regulate cell division. For example, addition of exogenous OGF or an increase in the number of its receptors downregulates cell division. Conversely, one can increase cell division by decreasing the interaction of OGF with its receptor, either by decreasing the production of OGF or its receptor or by utilizing a blocking agent, like the strong opioid antagonist, naltrexone (NTX), to directly block OGF-OGFr interaction.

**Figure 3:**
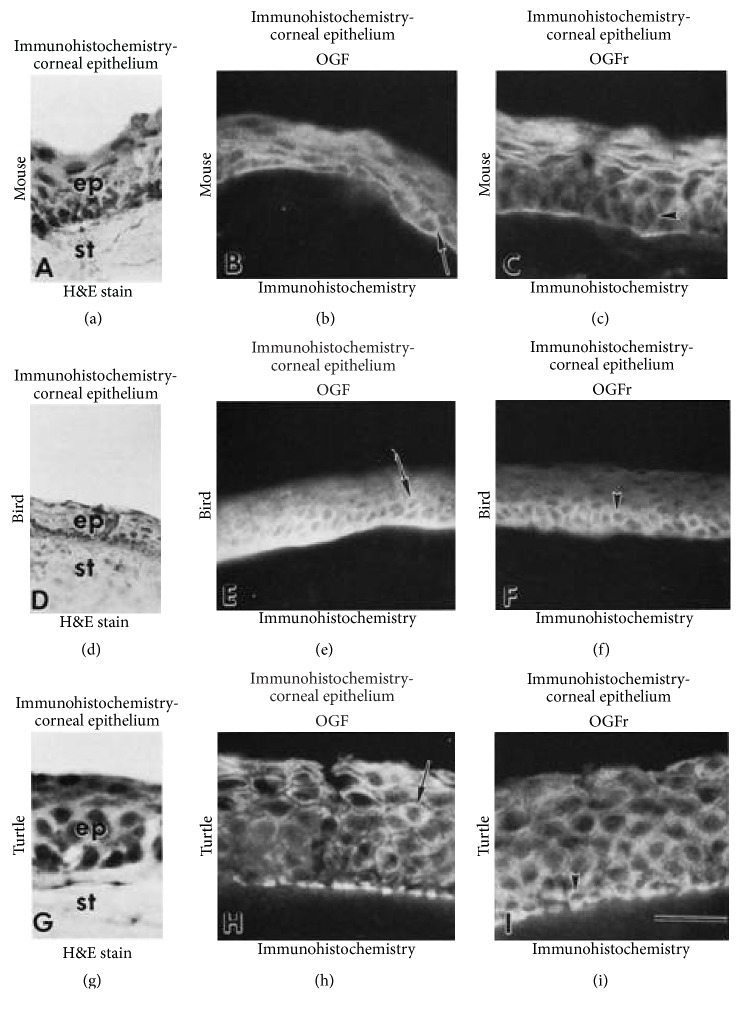
Immunohistochemistry for OGF and OGFr in mouse (a–c), bird (d–f), and turtle (g–i) corneal epithelium using brightfield (a, d, g) or indirect (b, c, e, f, h, i) immunofluorescence. Insets (a, d, g) are stained with hematoxylin and eosin and demonstrate the corneal epithelium (ep) containing basal and suprabasal cells with the underlying stroma (st). Immunofluorescence microscopy of tissue stained with anti-[Met^5^]-enkephalin (OGF) IgG (b, e, h) demonstrates OGF in the epithelial cortical cytoplasm of basal and suprabasal cells (arrows). Immunofluorescence of tissues stained with OGFr IgG (arrowheads in c, f, i) (derived from [5]).

The presence of the Opioid Growth Regulatory System has been demonstrated in the corneal epithelium of all vertebrate orders including mammals, birds, reptiles, amphibians, and fish, some of which are demonstrated in Figure 3 [5].

Over the past 25 years, the authors' research team has delineated the role of the Opioid Growth Regulatory System in the homeostasis and healing of ocular tissues. More recently, as will be discussed shortly, it has been shown to play a role in the pathobiology of diabetic ocular complications, such as depressed epithelial wound healing, abnormal corneal sensitivity, and dry eye, and in the nonocular complication of delayed healing of diabetic cutaneous wounds.

## 3. Corneal Epithelial Growth Regulation

In the corneal epithelium, OGF appears to be produced in an autocrine manner. For example, immunohistochemical examination of the corneal epithelium in the peripheral cornea, limbus, and conjunctiva has demonstrated the presence of preproenkephalin, the precursor to OGF, within the corneal epithelium in these regions thereby supporting the autocrine production of OGF by the corneal epithelial cells [6].

### 3.1. Homeostatic Corneal Epithelium: Nonhuman

**Figure 4:**
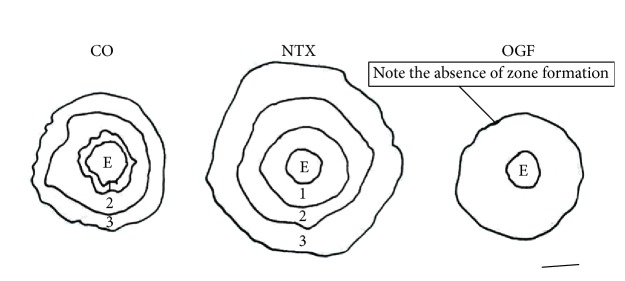
Outgrowths of corneal epithelium from corneal explants in organ culture from camera lucida drawings. CO: control; NTX: naltrexone supplemented culture media; OGF: opioid growth factor supplemented media; E: explant; numbers indicate zones of cellular outgrowth from the explant with zones 1 and 3 having active DNA synthesis and mitotic activity and zone 2 lacking DNA synthesis and mitotic activity. Note: explants exposed to media supplemented with OGF lack defined zone formation (derived from [7]).

As seen in Figure 4, corneal explants in culture demonstrate that the Opioid Growth Regulatory System modulates the outgrowth of homeostatic corneal epithelium, with exogenous OGF retarding and disorganizing the outgrowth and cell division of the epithelium and NTX accelerating outgrowth in reference to control explants without altering the normal outgrowth pattern [7, 8].

**Figure 5:**
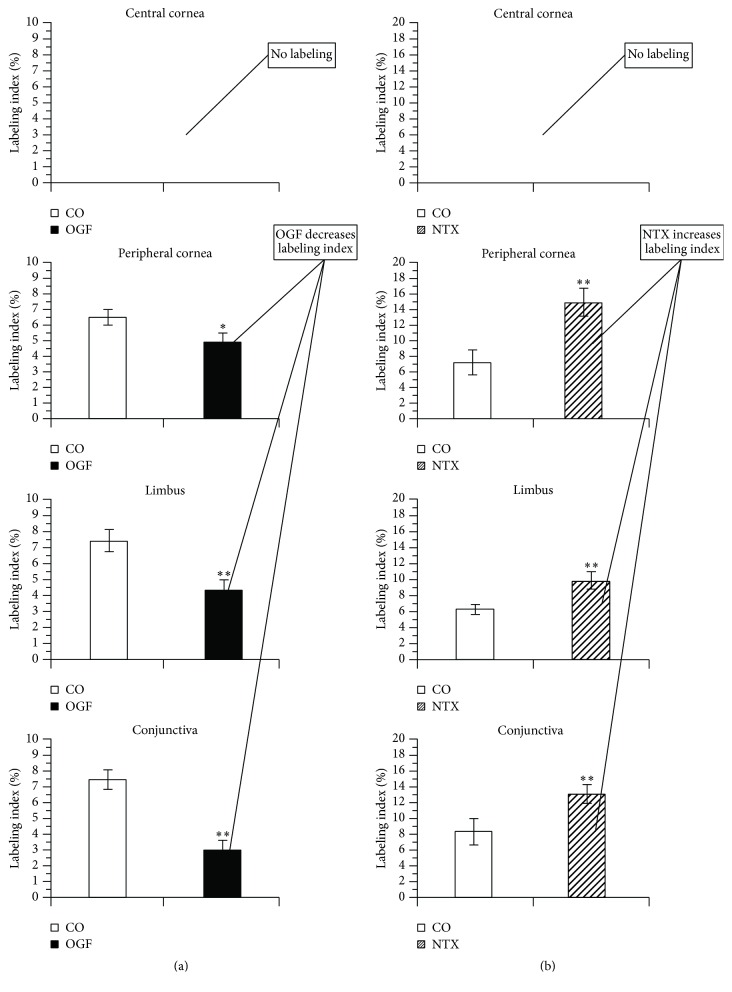
*In vivo* rat cornea: effects of OGF and NTX. Labeling index of basal corneal epithelium in the central cornea, peripheral cornea, limbus, and conjunctiva. Left series of histograms: OGF decreases labeling index of homeostatic rat corneal epithelium. Right series of histograms: NTX increases labeling index of homeostatic rat corneal epithelium. Note: neither OGF nor NTX has any effect on the central corneal epithelium (right and left, top graphs), which is postmitotic. ^*∗*^ *P* < 0.05 or ^*∗∗*^ *P* < 0.01 (derived from [8]).

If the Opioid Growth Regulatory System can modulate corneal epithelial migration and cell division in tissue culture, what is its impact on homeostatic corneal epithelium* in vivo*? Figure 5 documents the ability of treatment with OGF to suppress DNA synthesis in the cornea of the living rat. Conversely, NTX treatment significantly increases DNA synthesis [8]. (Please note that all findings or data cited in this review are significant at a minimum of *P* < 0.05, but, for the sake of brevity, no specific significance values will be presented except as cited in figures and their captions.)

### 3.2. Epithelial Wound Healing: Nonhuman

**Figure 6:**
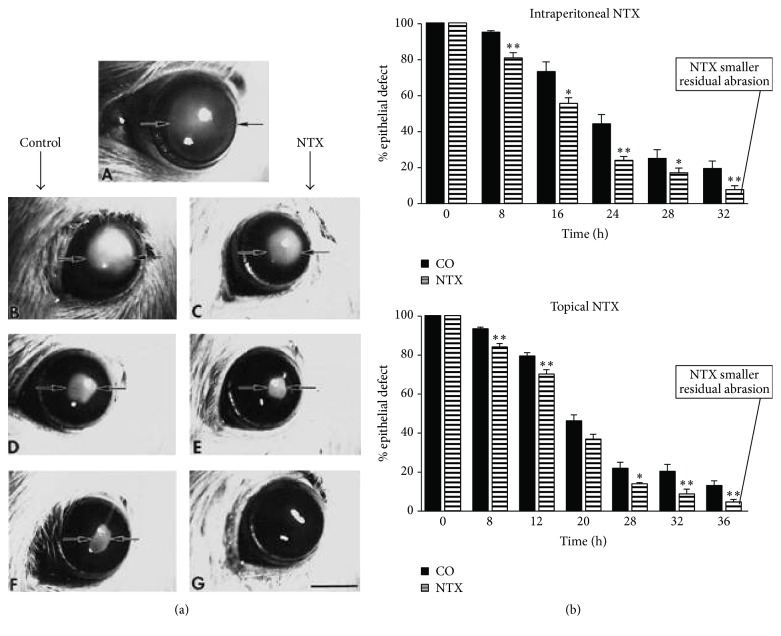
Rat cornea wound healing. (a) Photographs of rat corneas with 4 mm central abrasions stained with fluorescein immediately (A) or at 16 h (B, C), 24 h (D, E), or 32 h (F, G) after wounding and treated either with 30 mg/kg intraperitoneal injections of NTX (C, E, G) or with an equivalent volume of vehicle (B, D, F). (b) Histograms illustrate that both intraperitoneal injections of 30 mg/kg NTX or topical 10^−6^ M NTX eyedrops four times daily expedite rat corneal epithelial wound healing (derived from [10]). ^*∗*^ *P* < 0.05 and ^*∗∗*^ *P* < 0.01.

If blockade of the Opioid Growth Regulatory System positively impacts epithelial outgrowth in tissue and organ culture and increases DNA synthesis* in vivo,* how would it impact corneal epithelial wound healing? Indeed, treatment with either systemic or topical NTX results in an increased rate of rat corneal epithelial wound healing [9–11]. As illustrated in Figure 6, either intraperitoneal or topical NTX significantly increases the rate of reepithelialization of standardized rat corneal epithelial wounds. Similarly, rabbit corneal epithelial wound healing also is increased by blockade of the Opioid Growth Regulatory System by topical NTX [9–11].

### 3.3. Gene Transfer and OGFr

**Figure 7:**
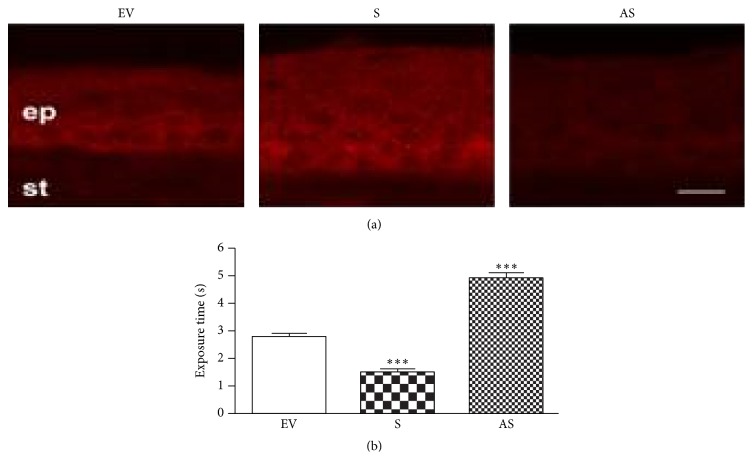
OGF-OGFr and molecular interactions: Gene Gun, Helios® Gene Gun System (Bio-Rad), used for particle-mediated gene transfer, which successfully delivers sense and antisense OGFr cDNA into corneal epithelial cells. (a) Fluorescence photomicrographs of immunohistochemical staining of peripheral cornea for OGFr expression. Sense (S) results in overexpression of OGFr. Antisense (AS) results in diminished expression of OGFr. Empty vector (EV) has no effect. (b) Histograms illustrate immunoreactivity response to particle-mediated transfection with empty vector (EV), sense (S), or antisense (AS). ^*∗∗∗*^ *P* < 0.001. Note: the *y*-axis is exposure time so an increased exposure time required for detection of the OGFr indicates a decreased amount of OGFr present in the sample (derived from [13]). All stained with OGFr antibodies.

Using the “Gene Gun,” one can specifically determine the role of the interaction of OGF and its receptor (OGFr) in regulating epithelial wound healing by delivering sense or antisense OGFr cDNA into corneal epithelial cells (Figure 7) [12, 13]. Sense cDNA increases OGFr production and antisense suppresses OGFr production. Overexpression of OGFr results in delayed wound healing of rat corneal epithelial abrasions and suppression of OGFr production using antisense cDNA results in expedited wound healing.

### 3.4. Lack of Toxic Effects of NTX Treatment

**Figure 8:**
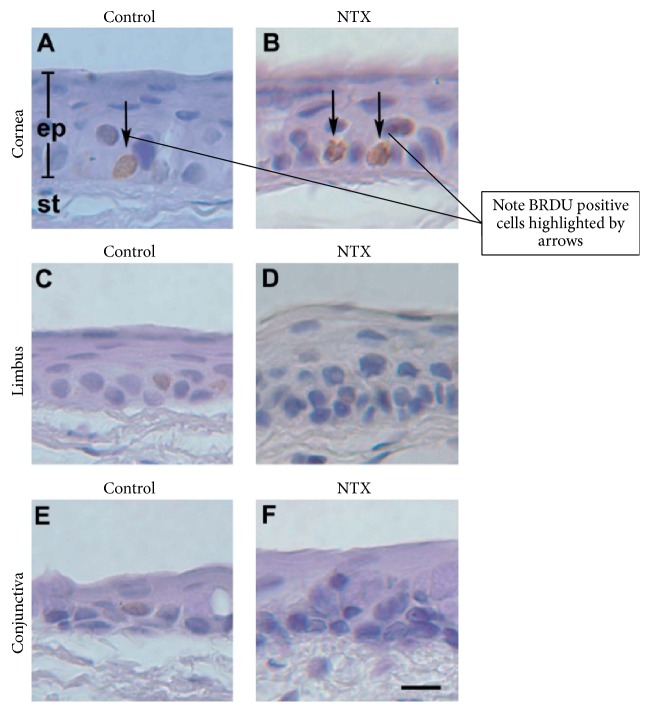
Impact of chronic NTX on homeostatic corneal epithelium. Seven-day treatment with systemic NTX. Rate of DNA synthesis determined using BrdU. Arrows are BrdU-positive, DNA synthetic cells. DNA synthetic cells increased by 69–85% following NTX treatment. Epithelial thickness increased by 8 to 38%. There was increased packing density. Nevertheless, no toxicity or proliferative pathology was seen. Rather, NTX treatment accelerates normal homeostatic processes, but there is negligible apoptosis or necrosis. st: stroma; ep: epithelium. Bar = 12 *µ*m (derived from [14]).

Is the increased corneal epithelial wound healing that is achieved through manipulation of the Opioid Growth Regulatory System accompanied by proliferative abnormalities in the epithelium? In order to answer this question, animals were treated* in vivo* for one week with NTX [8, 14].  Figure 8 demonstrates that DNA synthetic cells increased by 69–85% in response to NTX treatment. Epithelial thickness also increased by 8 to 38%. Cellular packing density was increased; however, no toxicity or proliferative pathology was seen. Rather, NTX treatment accelerates normal homeostatic processes. There was negligible apoptosis or necrosis.

### 3.5. Healing Corneal Epithelium: Human

**Figure 9:**
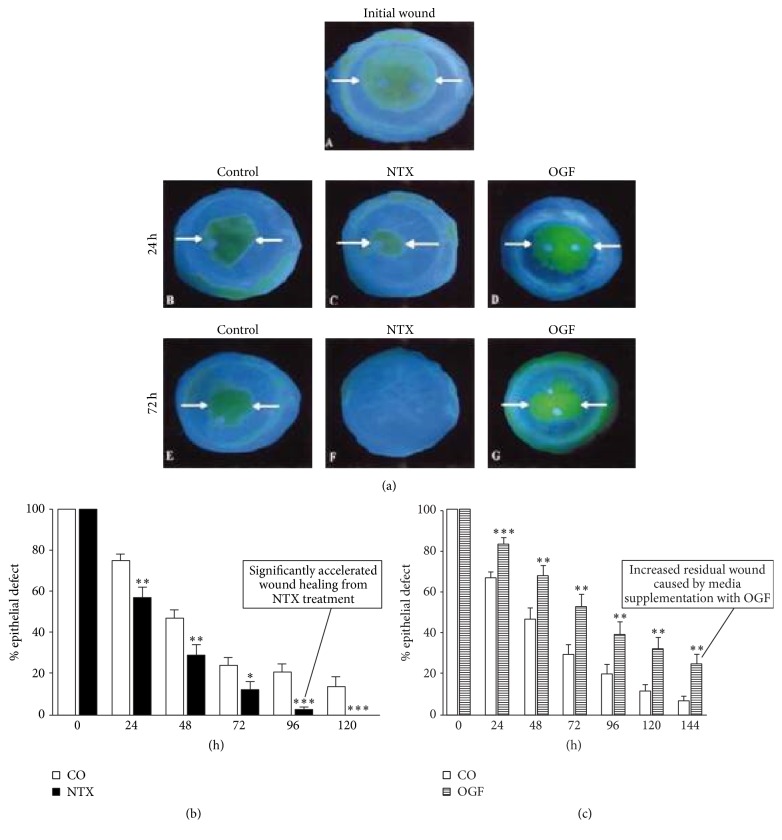
Human corneas in organ culture. (a) Photographs of abraded human corneas in organ culture at time of initial wounding and 24 and 72 hrs after wounding. Media supplemented with either sterile water (control) (B, E), 10^−6^ M NTX (C, F), or 10^−6^ M OGF (D, G). Note the more rapid healing response in NTX supplemented medium, compared to control, and decreased rate of wound healing with OGF medium supplementation. Arrows indicate the margins of the residual wounds. (b) Histograms illustrate the accelerated rate of epithelial wound healing in response to NTX culture medium supplementation. Overall, the healing of epithelial wounded human corneas cultured in medium supplemented with 10^−6^ M NTX was accelerated by 21% to 89% during the period of 24 to 96 hours. (c) Supplementation of culture medium with OGF suppresses epithelial wound healing. Histogram of residual epithelial defect in organ cultured human corneas following 8 mm epithelial wounds. Medium was supplemented with either sterile water (CO) or 10^−6^ M OGF. 24% to 260% more defects present in OGF-treated corneas at day 7. Data are expressed as means ± SEM. Significantly different from controls at ^*∗*^ *P* < 0.05, ^*∗∗*^ *P* < 0.01, or ^*∗∗∗*^ *P* < 0.001 (derived from [1]).

Just as the Opioid Growth Regulatory System regulates epithelial wound healing in animals, studies of organ cultured human corneas subjected to epithelial wounds demonstrated its impact on human epithelial wound healing.  Figure 9 illustrates accelerated human corneal epithelial healing of organ cultured corneas grown in culture medium supplemented with 10^−6^ M NTX. Conversely, supplementation of culture medium with OGF suppresses epithelial wound healing (Figure 9) [1].

Finally, OGF and NTX impact cultured human corneal DNA synthesis as one might anticipate with increased synthesis resulting from NTX treatment and DNA synthesis suppression from OGF supplementation.

## 4. Diabetes and the Opioid Growth Regulatory System

So what does all this have to do with diabetes?

### 4.1. Background

Diabetes is the leading cause of blindness among working-age adults in the United States [15]. In 2012, 29.1 million Americans or 9.3% of the population had diabetes. The prevalence of diabetes rises to 25.9% of American seniors [16].

Among the complications of diabetes is keratopathy. Both type 1 diabetes and type 2 diabetes are associated with keratopathy that is reflected in delayed corneal epithelial wound healing [17–19], abnormal corneal sensitivity [18–22], and dry eye [20, 21, 23, 24]. Unfortunately, none of the current treatments for these complications is uniformly effective [17].

Elevated levels of OGF, [Met^5^]-enkephalin, have been found in the plasma of diabetic patients [25–27]. Elevated OGF levels also have been found in genetically obese diabetic (db/db) mice, which are used as a model for type 2 diabetes [28–30]. Moreover, OGF and OGFr have been found in the corneal epithelium in diabetic animals [31]. It was postulated that abnormalities of opioid regulation could contribute to the complications of diabetes and that blockade of the Opioid Growth Regulatory System by NTX might reverse or ameliorate these complications.

The relevance of the Opioid Growth Regulatory System to the following diabetic corneal complications: delayed epithelial wound healing, abnormal corneal sensitivity, and dry eye will be discussed separately.

### 4.2. Diabetic Keratopathy

During the course of their disease, seventy percent of diabetics will suffer from diabetic keratopathy, which includes recurrent erosion, delayed wound healing, edema, and even ulcers [32–34]. These complications may occur spontaneously [35] or follow specific insults, such as ocular surgery [36–39].

Immunocytochemistry confirms the presence of OGF and OGFr in the corneal epithelium of diabetic animals.

**Figure 10:**
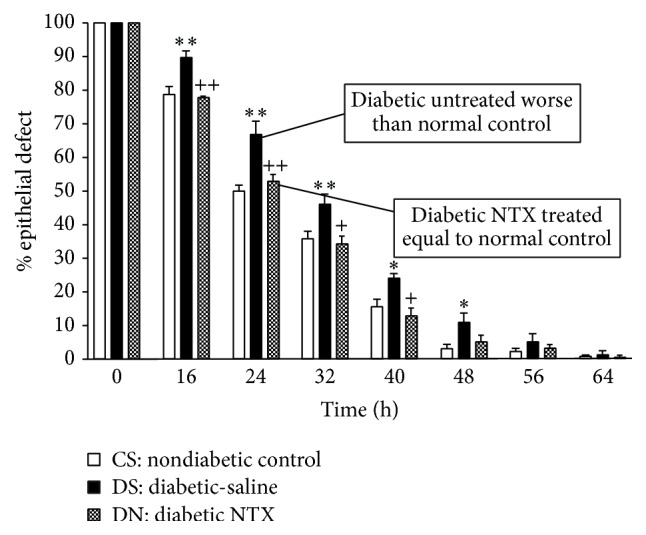
Naltrexone treatment normalizes epithelial wound healing in diabetic rats. Residual epithelial defect up to 64 h after creation of a 5 mm corneal abrasion. DS (untreated diabetic) had significantly larger residual epithelial defects than the CS (nondiabetic) or DN (NTX-treated diabetic rats). Therefore, NTX treatment prevents the delay in wound healing of ocular surface epithelium observed in poorly controlled diabetic animals. Data are expressed as means ± SEM and represent the residual of the original defects presented as a percentage of the original wounds. Significant difference between the DS and CS groups, ^*∗*^ *P* < 0.05 or ^*∗∗*^ *P* < 0.01. Significant difference between the DS and DN groups, ^+^ *P* < 0.05 or ^++^ *P* < 0.01 (derived from [31]).

In order to determine whether blockade of the Opioid Growth Regulatory System would improve epithelial wound healing in diabetes, standardized corneal epithelial wounds were produced in rats after four weeks of induced diabetes. Treatment with intraperitoneal NTX twice daily resulted in a marked increase in the rate of corneal reepithelialization compared to untreated control animals [31].  Figure 10 illustrates the impact of NTX treatment on the rate of corneal epithelial healing in untreated diabetic rats. Untreated diabetic animals healed at a rate that was significantly worse than that in normal controls. On the other hand, diabetic NTX-treated animals healed at a rate equal to that of the normal controls. Therefore, having demonstrated the ability of NTX to increase cell proliferation, it is not surprising that NTX treatment increased DNA synthesis in unwounded diabetic rat corneas 4-fold in the basal epithelium of the peripheral cornea, 3.5-fold in the limbal region, and 8-fold in the conjunctiva compared to control animals [31].

Does glucose control improve epithelial wound healing in diabetic rats and if so does NTX have an insulin-like effect on corneal epithelial wound healing?

In order to answer these questions, corneal epithelial wound healing in untreated diabetic rats was compared to that in animals treated with insulin minipumps [40]. At 40 hrs after wounding, untreated diabetic (DB) rats had significantly larger residual epithelial defects than the controls (either nondiabetic or DB-insulin-treated rats). This and other studies demonstrated clearly that intensive therapy with insulin, leading to normoglycemia in rats with diabetes, does prevent the delay in wound healing of ocular surface epithelium observed in poorly controlled diabetic animals.

Given that systemic control of diabetes facilitates corneal epithelial wound healing, does topical insulin have an effect independent of systemic glucose control?

In rats that have been diabetic for 9 or 11 weeks, topical insulin was administered four times daily for 7 days to wounded corneas. Diabetic animals treated with vehicle alone had wounds that were 35% larger than those in healthy vehicle-treated animals [40, 41]. Topical insulin treatment resulted in epithelial wounds that were 19% to 60% smaller than diabetic vehicle-treated ones. There was no insulin effect on healthy rat epithelium, and there was no effect on corneal thickness, IOP, apoptosis, or serum glucose levels. Thus, topical insulin treatment is effective in reversing the delayed epithelial wound healing characteristic of diabetic animals.

What is the effect of NTX treatment on corneal epithelial wound healing in diabetic animals?

**Figure 11:**
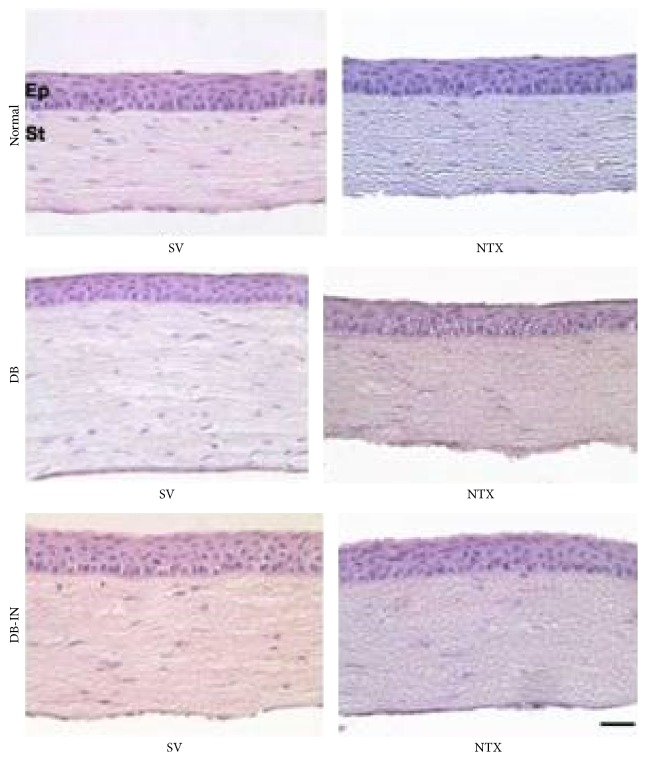
Toxicity evaluation of NTX-treated rat cornea. Corneal thickness, endothelial cell number, and evidence of epithelial apoptosis, necrosis, or organization were determined for topical 10^−3^ to 10^−7^ M NTX administered q.i.d. × 7 days to corneas of type 1 diabetic rats. Photomicrographs of histological sections of the rat peripheral cornea stained with hematoxylin and eosin from animals in normal, diabetic (DB), and diabetic-insulin (DB-IN) groups at 2 weeks after the conclusion of 7-day exposure (4 times daily) of 10^3^ M naltrexone (NTX) or sterile vehicle (SV). The morphology of the cells and tissues was similar between groups, and no pathologic changes were detected in corneas exposed to vehicle or 10^3^–10^7^ M NTX (data not shown for treatment with 10^−4^, 10^−5^, 10^−6^, or 10^−7^ M NTX). Ep: epithelium; St: stroma. Bar = 40 m (derived from [42]). These photomicrographs are of rat peripheral cornea treated with the topical 10^−3^ M NTX administered q.i.d. × 7 d or controls.

In preparation for testing NTX treatment for epithelial defects, a toxicity study of topical NTX was performed in insulin-controlled diabetic rats (Figure 11) [42]. There was no difference from normal rats or insulin-treated diabetic controls in IOP, corneal thickness, endothelial cell number, or epithelial apoptosis, necrosis, or organization. There was no overt toxicity of NTX over a 10,000-fold range of dosage.

Similarly, topical NTX proved as effective as intraperitoneal treatment for more rapidly healing epithelial defects in diabetic animals, and topical insulin was equally effective and safe as topical NTX for this purpose [40, 41, 43].

Combining topical insulin and topical NTX was not more effective than either one used independently [40, 41, 43, 44]. In short, there is a possibility that insulin and NTX have their effect through similar mechanisms in diabetic animals or that each medication has the potential to maximize epithelial wound healing in these animals, leaving no opportunity for further increase by the complementary modality. Furthermore, insulin has no effect on epithelial proliferation in normal animals, and NTX has no impact on blood glucose levels in diabetic animals.

**Figure 12:**
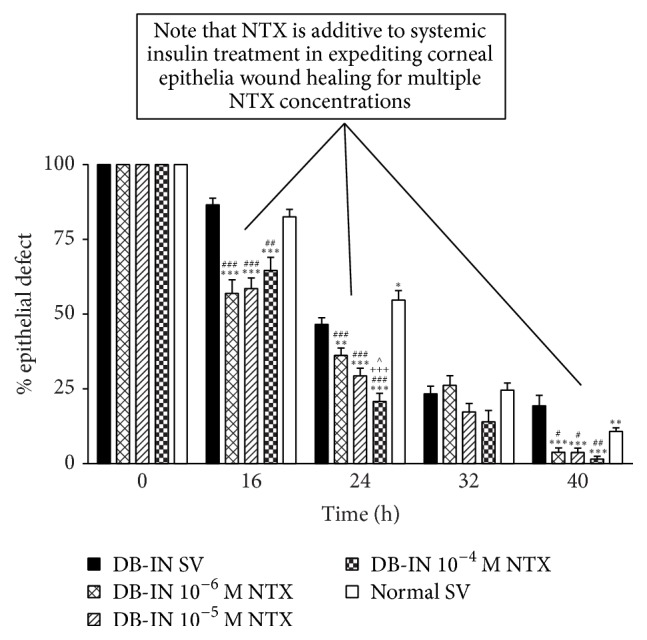
The figure compares the effectiveness of systemically administered insulin and topical NTX. Histograms demonstrate that topical NTX is additive to systemic insulin in facilitating epithelial wound healing in diabetic rats. Residual epithelial defects are presented as percentage of the original wound. Data expressed as means ± SEM. Significantly different from DB-IN rats receiving SV at ^*∗∗*^ *P* < 0.01 or ^*∗∗∗*^ *P* < 0.001 and from normal SV rats at ^#^ *P* < 0.05, ^##^ *P* < 0.01, or ^###^ *P* < 0.001. At 24 hrs, the 10^−4^ M NTX DB-IN group differed from the 10^−5^ M NTX DB-IN group at ^∧^ *P* < 0.05 and from the 10^−6^ M NTX DB-IN group at ^+++^ *P* < 0.001 (derived from [44]).

These data were compared to that involving the healing of corneal epithelial wounds in untreated or systemic insulin-treated diabetic rats given topical NTX (Figure 12). In both treatment groups, topical NTX significantly increased the rate of epithelial wound healing in contrast to the situation when insulin and NTX are combined in topical administration. One possible explanation for the difference in results obtained relative to the route of insulin administration (systemic versus topical) may be the inability of systemic insulin to reach the tear film in a concentration equivalent to that obtained with topical administration.

One should note that although NTX has been discussed relative to induced type 1 diabetes, it also is effective in facilitating corneal epithelial wound healing in obese db/db mice with type 2 diabetes on a genetic basis [45].

The potential toxicity of NTX applied topically four times daily for 7 days in concentrations of 10^−3^ to 10^−7^ M was evaluated* in vivo* in intact and abraded corneas of insulin controlled or uncontrolled diabetic rats [44]. Ocular surface morphology, intraocular pressure, corneal thickness, and corneal sensitivity were evaluated. Histopathologic studies were performed for apoptosis, necrosis, and endothelial cell counts. No toxicity from NTX treatment was found.

In summary, in diabetic animals, topical NTX restores corneal epithelial wound healing to levels comparable to systemically or topically insulin-treated animals without apparent epithelial toxicity.

### 4.3. Diabetic Corneal Neuropathy

Diabetic corneal neuropathy, particularly as assessed by confocal microscopy, correlates with peripheral neuropathy [46–52]. Corneal aesthesiometry (measuring corneal sensitivity to touch using progressively stiffer filaments, which are von Frey hairs) also can be helpful in the clinical assessment of diabetic corneal neuropathy [20, 50, 53, 54]. Diabetic corneal neuropathy is accompanied by delayed epithelial wound healing as described previously [55]. Corneal nerve damage can be induced by obesity related to diet or to type 2 diabetes [56]. Moreover, diabetic corneal nerve injury is repairable as evidenced by the fact that corneal nerve regeneration has been demonstrated after simultaneous kidney and pancreas transplantation [57] and other therapies [58].

**Figure 13:**
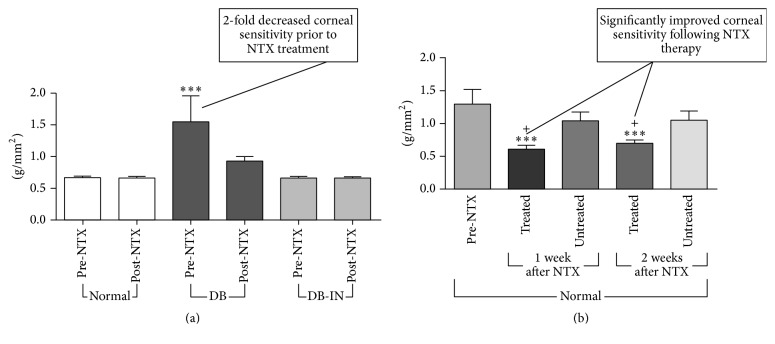
Impact of NTX treatment on corneal sensitivity. (a) Prior to NTX treatment: 2-fold decreased corneal sensitivity in the 8-week DB untreated diabetic rats compared to normal controls (increased force required to generate a response). (b) After NTX treatment, DB sensitivity returned to normal levels with a 2-fold and 1.5-fold increase in sensitivity, respectively, following NTX treatment. There was no effect on corneal sensitivity and there was no effect on normal or insulin treated diabetic (DB-In) rats. Note: in both histograms, higher bar is less sensitive. Values are means ± SEM. Significantly different from NTX at ^*∗∗∗*^ *P* < 0.001 and untreated corneas at ^+^ *P* < 0.05 (derived from [42]). Height of histogram indicates force required for stimulus to be detected. Higher histogram indicates less sensitivity.

In order to evaluate the reversibility of corneal diabetic neuropathy, rats having eight weeks of induced diabetes were treated with 1 or 5 days of four times daily NTX at 10^−5^ M concentration (Figure 13) [42, 59]. Corneal sensitivity was restored to normal levels beginning one hour after termination of drug exposure and extending for at least 4 days thereafter. Conversely, control diabetic animals maintained sensitivity scores that were 1.5- to 2.0-fold less than both the normal and NTX-treated groups.

The NTX effect resulting in normalization of corneal sensitivity ended after 120 hrs, for animals treated for one day, and 192 hrs following discontinuation of a 5-day treatment period with four times daily NTX. At those respective time points, corneal sensitivity reverted to being 1.9-fold less than normal animals and comparable to control diabetic animals. Thus, the period of normalcy only can be attributed to NTX therapy.

NTX also is effective in restoring corneal sensitivity to normal levels in obese db/db type 2 diabetic mice [45].

In summary, topical NTX treatment restores corneal sensitivity to normal levels in both type 1 and type 2 diabetic rats. These findings implicate the Opioid Growth Regulatory System in the pathobiology of diabetic corneal neuropathy and are consistent with other studies cited above, which suggest that diabetic corneal neuropathy can be reversible.

### 4.4. Dry Eye

Dry eye is more common in diabetic patients and correlates with poor glycemic control [21, 23, 60]. Moreover, diabetic dry eye is more common in individuals with diabetic retinopathy of increased severity [61].

**Figure 14:**
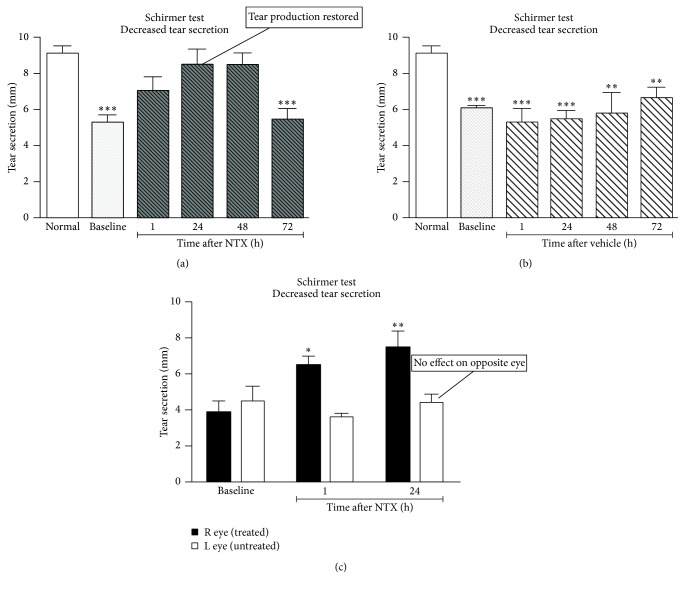
Impact of NTX treatment on tear secretion. (a) One drop of 10^−5^ M NTX results in tear levels returning to normal for up to 48 hr. (b) Vehicle alone results in no improvement in tear secretion. Significantly different from normal rats at ^*∗∗*^ *P* < 0.01 or ^*∗∗∗*^ *P* < 0.001. (c) Right and left eyes compared after one drop of 10^−5^ M NTX in the right eye. Significantly different from left eye at ^*∗*^ *P* < 0.05 and ^*∗∗*^ *P* < 0.01. Values represent means ± SEM (derived from Zagon et al., 2012 [62]).

Apparently normal rats have periods during which there is a spontaneous decrease in tear production (Figure 14) [62]. It was determined that one drop of 10^−5^ M NTX restores tear production to normal levels for up to 48 hrs in such animals. Vehicle alone results in no improvement in tear secretion. If right and left eyes are compared after one drop of 10^−5^ M NTX in the right eye, there is no effect on the contralateral eye.

Conversely, neither one drop of 10^−5^ M NTX nor vehicle had any impact on tear production that already was at a normal level. There was no difference in corneal sensitivity during periods of normal or reduced tear production over a 20-fold difference in force using von Frey hairs, which are used to test for corneal sensitivity to touch [62].

**Figure 15:**
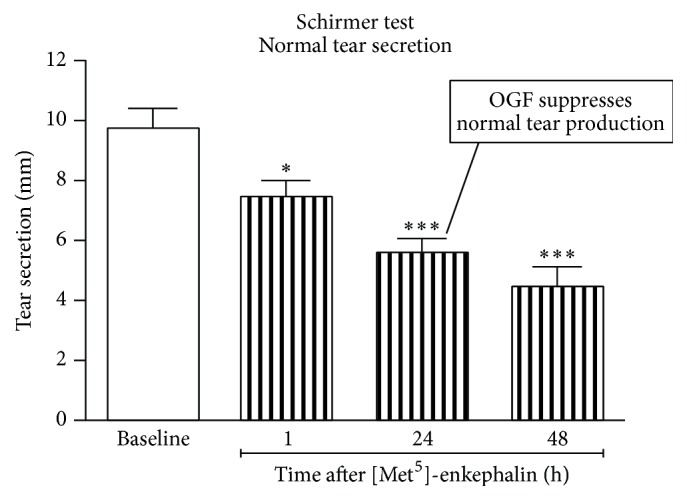
Impact of OGF treatment on tear secretion. One drop of 10^−5^ M OGF, [Met^5^]-enkephalin, significantly reduces tear production in rats with initially normal levels. Values represent means ± SEM (derived from [62]). ∗ refers to *P* < 0.05 and ∗∗∗ refers to *P* < 0.001.

Although NTX has no ability to raise tear production in rats with normal tear secretion, one drop of 10^−5^ M OGF, [Met^5^]-enkephalin, significantly reduces tear production in rats with initially normal levels (Figure 15) [62].

Thus, NTX blockade of the Opioid Growth Regulatory System appears to have the ability to raise tear production to normal levels in nondiabetic rats having a period of depressed tear production. Conversely, OGF can depress tear production to subnormal levels even in nondiabetic rats. These data support the concept of Opioid Growth Regulatory System modulation of tear production even in nondiabetic rats.

What is the impact of diabetes on tear production? Dry eye was evaluated in rats having type 1 diabetes of 8-week duration treated with four times daily topical NTX at 10^−5^ M concentration. Untreated diabetic rats had tear production reduced by 32% to 53% compared to normal or to NTX-treated animals. In contrast, diabetic rats treated with NTX had tear production similar to normal rats extending for at least 3 days following the termination of treatment. By 96 hours after termination of treatment, tear production had decreased again to 22% to 59% less than normal animals, thereby emphasizing how effective the previous NTX treatment had been.

**Figure 16:**
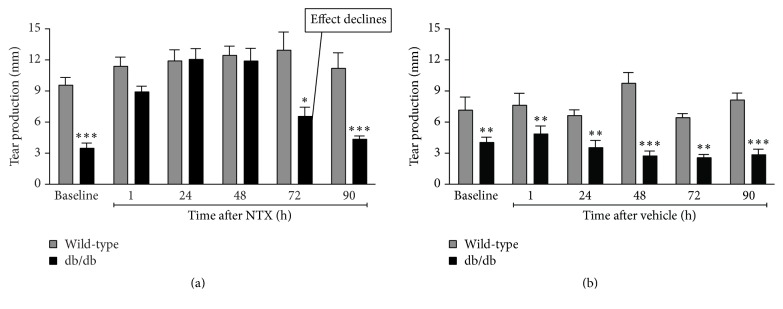
Tear production in wild-type or db/db mice given one drop of 10^−5^ M NTX (a) or only vehicle (b). (a) Note the rise in tear production to normal levels with NTX until about 72 hrs after treatment. No effect from vehicle alone (b) with abnormal tear production at all times tested. Ocular surface abnormalities related to type 2 diabetes are reversed by the opioid antagonist naltrexone. Significantly different from measurements for corresponding wild-type mice at each time point at ^*∗*^ *P* < 0.05, ^*∗∗*^ *P* < 0.01, and ^*∗∗∗*^ *P* < 0.001 (derived from [45]).

Figure 16 illustrates tear production in wild-type or db/db type 2 diabetic mice given one drop of 10^−5^ M NTX (a) or only vehicle (b). Note the rise in tear production to normal levels with NTX treatment until about 72 hrs after treatment (a). There was no effect from vehicle alone (b) on abnormal tear production at all times tested [45].

In summary, these findings support a role for Opioid Growth Regulatory System in the pathobiology of abnormal diabetic tear production in that NTX treatment restores tear production to normal levels for up to 3 days following discontinuation of the therapy in type 1 or type 2 diabetic rats.

### 4.5. Other Diabetic Complications: Cutaneous Ulcers

The research reported above demonstrates that the Opioid Growth Regulatory System is important in the pathobiology of diabetic ocular surface disease, in that manipulating the system through blockade of the OGFr with NTX restores corneal epithelial wound healing, ocular sensation, and tear production to normal levels in diabetic animals. Nevertheless, the question arises as to whether these findings are only of localized importance restricted to the ocular region or whether they are manifestations of a systemic abnormality of the Opioid Growth Regulatory System secondary to diabetes.

Delayed or incomplete healing of cutaneous wounds, particularly foot ulcers, is an important systemic diabetic complication. For example, diabetic foot ulcers are said to be one of the most costly and devastating complications of diabetes mellitus and affect 15% of diabetic patients during their lifetime [63]. Therefore, the impact of Opioid Growth Regulatory System blockade on cutaneous wound healing in diabetic rats was evaluated to test the effectiveness of NTX on this important and quantifiable indicator for the systemic complications of diabetes.

**Figure 17:**
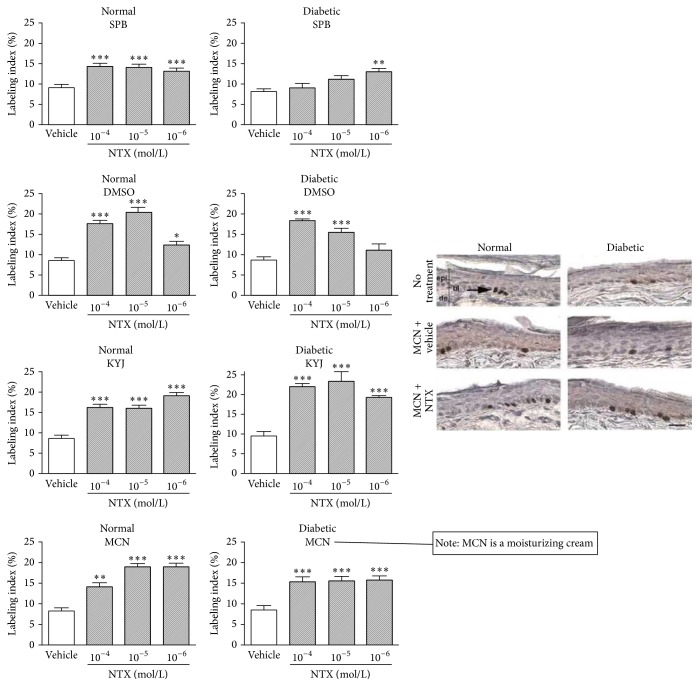
Effect of topical NTX in various vehicles on labeling index (LI) of skin epithelium. NTX was applied topically to the skin in type 1 diabetic rats or normal rats using NTX 10^−4^ M, NTX 10^−5^ M, or NTX 10^−6^ M in Sorenson's phosphate buffer, lubricant, moisturizing cream, or dimethylsulfoxide. NTX applied TID topically to dorsal skin of normal rats elevated LI by 43% to 132% in any of the four vehicles compared to normal baseline. NTX applied TID topically to dorsal skin of DB rats elevated LI by 103–147% in lubricant and by 85–89% in moisturizing cream. Photomicrographs demonstrate relative frequency of DNA synthesizing cells containing label. SPB: buffered saline; DMSO: dimethylsulfoxide; KYJ: sterile lubricating jelly; MCN: moisturizing cream (derived from [64]). ^*∗*^ *P* < 0.05, ^*∗∗*^ *P* < 0.01, and ^*∗∗∗*^ *P* < 0.001.

The following research was performed by Jessica Immonen, a graduate student then and now Doctor Immonen, at the Department of Anatomy at Rocky Mountain University of Health Professions, as the basis for her Masters and Doctorate degrees. Working with Drs. Zagon and McLaughlin, from our research team, Dr. Immonen evaluated the impact of NTX 10^−4^ M, NTX 10^−5^ M, or NTX 10^−6^ M in Sorenson's phosphate buffer, lubricant, moisturizing cream, or dimethylsulfoxide applied to the skin surface three times daily on DNA synthesis in the skin of normal or type 1 diabetic rats (Figure 17). She discovered that cutaneous DNA synthesis in unwounded normal rats was elevated by 43% to 132% in response to NTX in any of the four vehicles compared to normal baseline. NTX applied three times daily topically to dorsal skin of DB rats elevated labeling index (LI) by 103–147% in lubricant and by 85–89% in moisturizing cream. Note the DNA synthesizing cells in the photomicrographs in Figure 17 [64].

**Figure 18:**
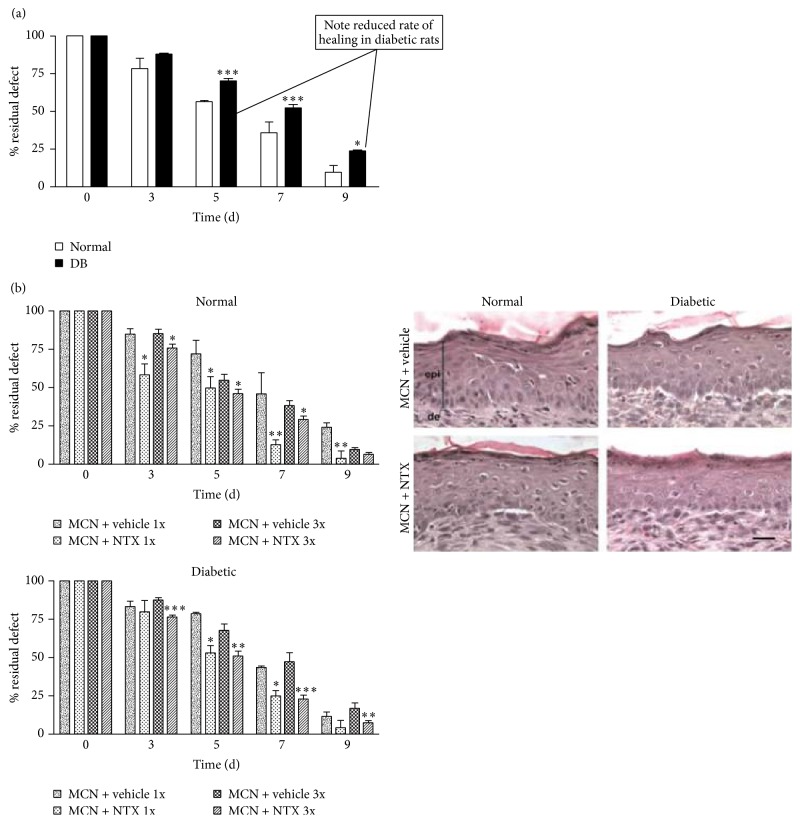
Effect of NTX treatment on wound healing rate of standardized skin wounds. Left: the impact of daily or three times daily NTX in moisturizing cream (MCN) on the healing rate of standard cutaneous wounds. Left (a): untreated diabetic rats had skin wounds that were 24%, 44%, and 132% greater than those of normal animals on days 5, 7, and 9, respectively, after wounding. Left (b, upper): within three days of a single or three times daily regimen of 10^−5^ M NTX in MCN, the wounded areas of normal rats were reduced by 30% and 11%, on the respective days, compared to other normal animals receiving MCN + vehicle. Left (b, lower): diabetic (DB) animals subjected to once daily application of MCN + NTX had a decreased wound size compared with DB rats exposed to vehicle, but the greatest effect was achieved with MCN + NTX given three times daily, which consistently accelerated wound closure, resulting in mean residual wounds that were reduced by 13% to 57% from DB rats treated with MCN + vehicle. Values represent means ± SEM. Significantly different from normal or MCN + vehicle at ^*∗*^ *P* < 0.05, ^*∗∗*^ *P* < 0.01, and ^*∗∗∗*^ *P* < 0.001. Right: histology of skin comparing normal and DB rats receiving vehicle or NTX. The overall appearance is similar, without evidence of necrosis, and so forth. Nevertheless, epithelial thickness in normal rats (24.6 ± 1.6 *μ*m) was 44% greater than in DB rats receiving vehicle. Normal animals treated with NTX, as well as DB rats subjected to NTX, did not differ in the thickness of the epithelium relative to normal animals receiving vehicle (derived from [64]).

A model of 6 mm full-thickness cutaneous wounds was used to investigate the healing response to one of the previously tested concentrations of NTX, 10^−5^ M, in either moisturizing cream (MCN) or vehicle alone (Figure 18). Within 3 days of treatment initiation, normal rats treated with once or three times daily NTX had wounds 30% and 11% smaller at the respective dosages than control animals. Diabetic animals treated with NTX in moisturizing cream had wounds 13% to 57% smaller than diabetic controls. There was no difference in skin histology between NTX-treated and control animals [64].

**Figure 19:**
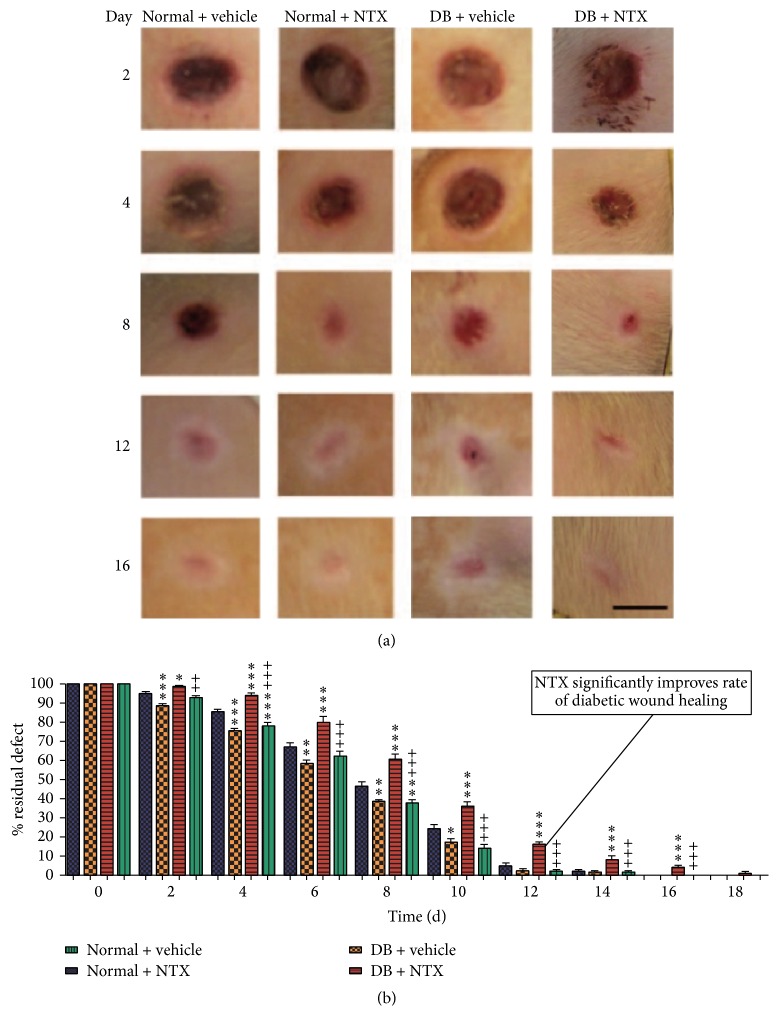
Full-thickness wound closure following application of topical NTX to normal and hyperglycemic rats. (a) Full-thickness wounds created on the dorsal surface of normal and type 1 diabetic rats. Wounds were treated three times daily with either 10^−5^ mol/L NTX (NTX) or saline (vehicle) dissolved in Neutrogena moisturizing cream. Photographs were taken every other day for 18 days. Bar = 5 mm. (b) Histograms of residual defects as a % of the original wounds. Groups were treated with either NTX or vehicle over an 18-day period of time. Diabetic NTX-treated rats (DB + NTX) had wounds 62–89% smaller compared to DB controls (DB + vehicle). Diabetic NTX-treated rats (DB NTX) had wounds 62–89% smaller than DB controls. Significantly different from normal + vehicle measurements at ^*∗*^ *P* < 0.05, ^*∗∗*^ *P* <  0.01, and ^*∗∗∗*^ *P* < 0.001; significantly different from DB + vehicle group at ^++^ *P* < 0.01 and ^+++^ *P* < 0.001. NTX: naltrexone; DB: diabetic (derived from [65]).

When normal (N) rats with standard skin wounds were treated three times daily with 10^−5^ M NTX in moisturizing cream, they healed 6–26% faster than normal control rats. Diabetic NTX-treated rats had wounds 62–89% smaller than diabetic controls (Figure 19) [65].

NTX appears to improve cutaneous wound healing, in part by stimulating angiogenesis. Diabetic control animals have delayed expression of fibroblast growth factor-2 (FGF-2) and vascular endothelial growth factor (VEGF). Conversely, topical NTX stimulates expression of these angiogenic factors. Similar findings are noted for the expression of *α*-smooth muscle actin (SMA) in capillaries [65].

In summary, topical NTX accelerates cutaneous wound closure, at least in part, by stimulating expression of angiogenic factors within healing tissue of diabetic animals. Obviously, there is the potential for NTX treatment to have a significant impact on facilitating the initial closure of such wounds in diabetic patients.

**Figure 20:**
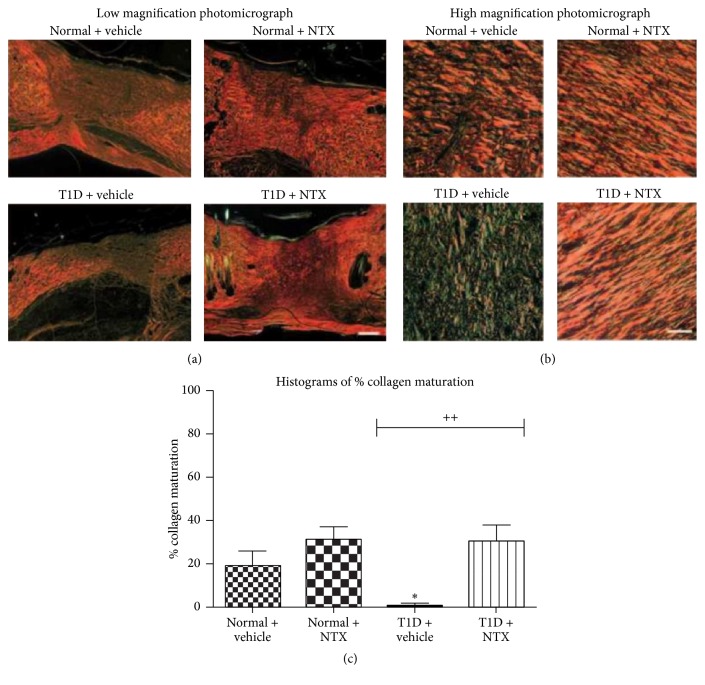
Sirius red-stained healing skin. Diminished collagen formation and maturation in diabetes are restored to normal by NTX treatment. These figures demonstrate remodeling of full-thickness wounds created in T1D and normal rats as measured by collagen formation in the reticular dermis 20 days after wounding and treatment with NTX dissolved in moisturizing cream or cream alone (vehicle). Wounds from normal and T1D rats were treated three times daily with either 10^−5^ M NTX or sterile saline (vehicle) dissolved in moisturizing cream. (a) Low magnification (4x) photomicrographs of Sirius red birefringence of skin sections encompassing the full-thickness wound and peripheral unwounded skin; bar = 100 mm. (b) High magnification of collagen maturation in Sirius red-stained sections described in (a); bar = 25 mm. (c) Histograms of the percent collagen maturation analyzed by ImageJ at 20 days. Values represent means ± SEM. ^*∗*^Significantly different from normal + vehicle values at *P* < 0.05; ^++^significantly different between T1D + vehicle and T1D + NTX at *P* < 0.01 (derived from [66]).

NTX has a more pervasive impact on the overall process of wound healing beyond just wound closure. For example, birefringence of Sirius red-stained healing granulation tissue revealed increased collagen formation and maturation in NTX-treated animals (Figure 20) [66].

Finally, inadequate wound healing at 60 days after wounding in diabetic control animals is further demonstrated by reduced tensile strength in comparison to control nondiabetic animals or to NTX-treated diabetic wounds, which have tensile strength similar to normal controls [66].

**Figure 21:**
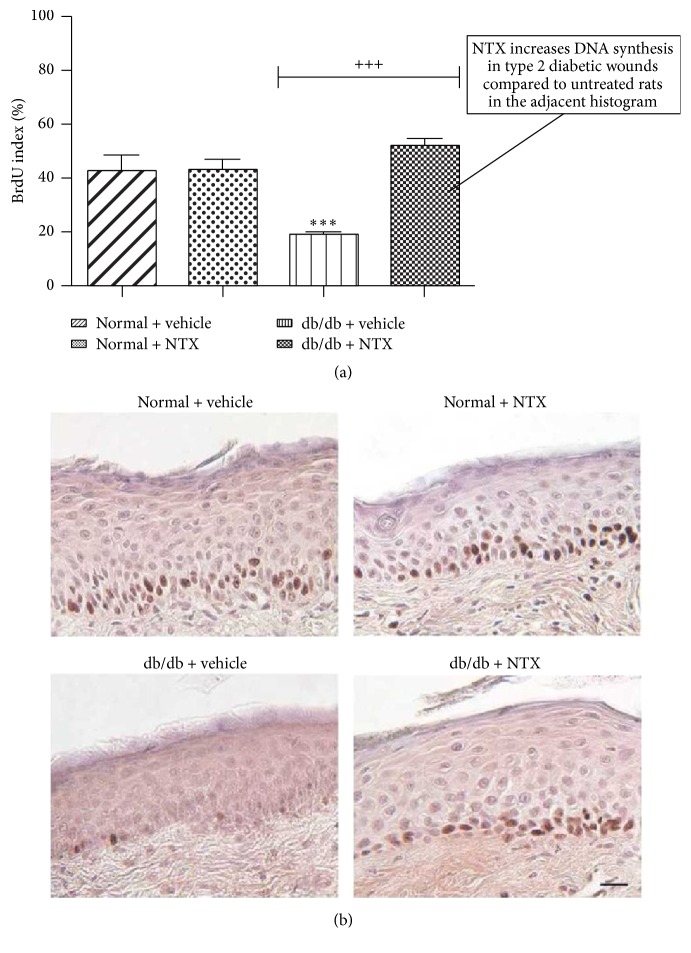
Spontaneously diabetic db/db mice with 5 mm full-thickness skin wounds on dorsum were treated with topical 10^−5^ M NTX in moisturizing cream or saline dissolved in moisturizing cream TID × 14 days. (a) DNA labeling indices calculated from the number of BrdU-positive basal cells relative to the total number of basal cells. Depressed DNA synthesis is noted in the DB rats compared to normal or NTX-treated DB animals. NTX-treated DB mice had BrdU LI of 52% compared to 43% for normal and NTX-treated normal animals. Values represent mean ± SEM. Significantly different from normal + vehicle group at ^*∗∗∗*^ *P* < 0.001. Labeling indexes in NTX-treated diabetic (db/db + NTX) skin were significantly different from measurements in saline-treated tissue from T2D mice (db/db + vehicle) at ^+++^ *P* < 0.001. (b) Sections of the skin treated as above illustrate the relative labeling amounts of the treatment groups (derived from [67]).

**Figure 22:**
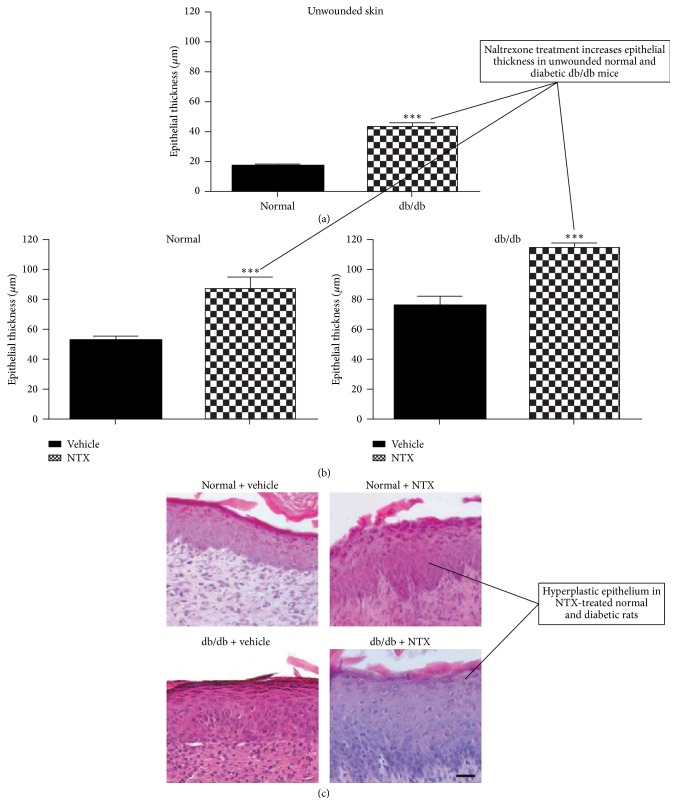
Histograms and photomicrographs illustrate the impact of 14 days of treatment with topical application three times daily of NTX or saline dissolved in Neutrogena moisturizing cream on the epithelialization of cutaneous wounds in normal and diabetic (db/db) mice. (a) The epithelium is hyperplastic in the NTX-treated normal and DB mice as illustrated in the histogram of epithelial thickness in normal and db/db unwounded skin. Values represent means ± SEM. Significantly different from normal values at ^*∗∗∗*^ *P* < 0.001. (b) Histograms of epithelial thickness over the underlying connective tissue in the wound bed in normal and db/db wounded skin treated with NTX or saline dissolved in moisturizing cream (vehicle). Values represent means ± SEM. Data from NTX-treated wounds were compared with vehicle-treated wounds for normal or db/db mice. Significantly different from vehicle-treated specimens at ^*∗∗∗*^ *P* < 0.001. (c) Photomicrographs of epithelium stained with hematoxylin and eosin permit comparison of the epithelial thickness among normal, diabetic NTX-treated, and control epithelium; bar = 25 *µ*m (derived from [67]).

It must be noted that NTX treatment of standardized cutaneous wounds in the spontaneously diabetic db/db mouse model of type 2 diabetes also results in an increased labeling index and more rapid wound closure comparable to normal levels (Figure 21) [67]. It is interesting that, in these db/db mice, epithelium was hyperplastic in the skin of unwounded NTX-treated normal and DB rats compared to their counterparts (Figure 22) [67].

In summary, Opioid Growth Regulatory System blockade by NTX significantly and positively impacts cutaneous wound healing in diabetic animals thereby demonstrating its involvement in the pathobiology of systemic, nonocular diabetic complications.

## 5. Summary of Research and Implications

The Opioid Growth Regulatory System is a phylogenetically ancient growth regulatory system that has been conserved across multiple existing animal phyla and, specifically, in ocular tissue. It regulates cell division in all cell types capable of dividing that have been tested to date including normal, healing, embryologic, and neoplastic tissues.

The function of the Opioid Growth Regulatory System appears to be disordered in diabetic animals, and its function can be restored with NTX treatment to normalize corneal epithelial wound healing, corneal sensitivity, and tear production in models of both type 1 and type 2 diabetes. Moreover, studies by our team relative to cutaneous wound healing in diabetic animals further support the hypothesis that the Opioid Growth Regulatory System is disordered relative to wound closure, wound maturation, and the restoration of tissue tensile strength in nonocular tissue, specifically the skin. Thus, our findings support the hypothesis that the function of the Opioid Growth Regulatory System is diffusely and abnormally impacted by diabetes.

Where do we go from here?

Naltrexone has been shown to be well tolerated in short-term ocular application in healthy human volunteers [68]. Further studies leading to the topical use of NTX in wound healing are required. Moreover, in our opinion, a more global study, to determine the impact of NTX therapy on the prevention of systemic complications of diabetes, is indicated.
